# N-Doped Graphene with Low Intrinsic Defect Densities via a Solid Source Doping Technique

**DOI:** 10.3390/nano7100302

**Published:** 2017-09-30

**Authors:** Bo Liu, Chia-Ming Yang, Zhiwei Liu, Chao-Sung Lai

**Affiliations:** 1 State Key Laboratory of Electronic Thin Films and Integrate Devices, University of Electronic Science and Technology of China, Chengdu 610054, China; doctorliu8963@gmail.com (B.L.); ziv_liu@hotmail.com (Z.L.); 2 Department of Electronic Engineering, Chang Gung University, Taoyuan 33302, Taiwan; cmyang@mail.cgu.edu.tw; 3 Institute of Electro-Optical Engineering, Chang Gung University, Taoyuan 33302, Taiwan; 4 Biosensor Group, Biomedical Engineering Research Center, Chang Gung University, Taoyuan 33302, Taiwan; 5 Department of General Surgery, Chang Gung Memorial Hospital, Linkou 33305, Taiwan; 6 Department of Nephrology, Chang Gung Memorial Hospital, Linkou 33305, Taiwan; 7 Department of Materials Engineering, Ming Chi University of Technology, New Taipei City 24301, Taiwan

**Keywords:** solid source doping technique, CVD, N-doped graphene, low defects, field-effect transistors

## Abstract

N-doped graphene with low intrinsic defect densities was obtained by combining a solid source doping technique and chemical vapor deposition (CVD). The solid source for N-doping was embedded into the copper substrate by NH_3_ plasma immersion. During the treatment, NH_3_ plasma radicals not only flattened the Cu substrate such that the root-mean-square roughness value gradually decreased from 51.9 nm to 15.5 nm but also enhanced the nitrogen content in the Cu substrate. The smooth surface of copper enables good control of graphene growth and the decoupling of height fluctuations and ripple effects, which compensate for the Coulomb scattering by nitrogen incorporation. On the other hand, the nitrogen atoms on the pre-treated Cu surface enable nitrogen incorporation with low defect densities, causing less damage to the graphene structure during the process. Most incorporated nitrogen atoms are found in the pyrrolic configuration, with the nitrogen fraction ranging from 1.64% to 3.05%, while the samples exhibit low defect densities, as revealed by Raman spectroscopy. In the top-gated graphene transistor measurement, N-doped graphene exhibits n-type behavior, and the obtained carrier mobilities are greater than 1100 cm^2^·V^−1^·s^−1^. In this study, an efficient and minimally damaging n-doping approach was proposed for graphene nanoelectronic applications.

## 1. Introduction

Graphene is a promising next generation electronic material, and its electronic properties can be modified by the substitutional doping of heteroatoms (e.g., boron [1], nitrogen [2], fluorine [3], and sulfur [4]). Among these atoms, nitrogen, which forms stable covalent bonds due to its similar atomic size and introduces an additional n-type valance electron, is widely used as the heteroatom for adjusting graphene properties. Nitrogen substitution doping in the graphene lattice gives rise to potential applications in fuel cells [5], lithium ion batteries [6], molecular sensors [7], ultracapacitors [8], and nanoelectronics [9] because nitrogen atoms disrupt the intrinsic graphene hybridization and locally induce significant modifications to its charge distribution and chemical reactivity [10]. Furthermore, with nitrogen doping, the work function of graphene shifts to lower states [11], providing broad and adjustable band applications in graphene p-n junctions [12] and mixed-dimensional van der Waals structures [13] (2D-*i*D, *i* = 0, 1, 2, 3, e.g., graphene-quantum dots, graphene-carbon nanotubes (CNTs), graphene-2D materials, and graphene-bulk materials). To obtain these promising properties and applications, researchers have continuously endeavored to synthesis N-doped graphene without damaging the graphene crystal structure and deteriorating its carrier mobility.

The synthesis methods for N-doped graphene can be briefly classified into two main categories [14]: (i) post-treatment in which nitrogen is incorporated into graphene or graphene oxide by post-annealing in NH_3_ gas or by NH_3_ plasma [7,11,15,16,17] or via Ultraviolet illumination in NH_3_ [18] and (ii) direct doping during synthesis in which carbon/nitrogen precursors such as CH_4_/H_2_/NH_3_ [19] and pyridine [9] are simultaneously introduced during the graphene growth, specifically in chemical vapor deposition (CVD). Despite the achievements in the successful synthesis of N-doped graphene in which the graphene properties can be tailored and the crystal structure can be preserved, the incorporation processes are always accompanied by high defect densities and graphene carrier mobility degradation. For the transport mechanism, the presence of additional structural defects and the process of nitrogen incorporation activate short-range (intrinsic lattice defects) and long-range scattering (ionized impurities or ripples), respectively [20,21]. Therefore, the transport behavior of N-doped graphene is strongly dependent on its synthesis process, including the nitrogen doping process and the accompanying formation of defects.

To obtain N-doped graphene with low defect densities, Cory et al. determined the ion energy window for nitrogen doping in the graphene lattice with the balance between the substitutional reaction efficiency and the vacancies or defect formation [22]. Moreover, George et al. proposed a high-temperature (between 1150 to 1200 °C) post-annealing method for efficient nitrogen doping with negligible defect densities [23]. During the high-temperature post-annealing in NH_3_, covalent C–N bonds form at intrinsic defects, vacancies or sites with weak C–C bonds in which the carbon atoms can be easily replaced by nitrogen atoms. However, considering the mismatch of the thermal expansion coefficients between graphene and the underlying substrate and the electrical disturbance induced by the closer contact between graphene and the underlying substrate after high-temperature post-annealing, extensive research must be devoted to its transport performance after high-temperature post-annealing [24]. For the method of direct doping during growth, two main challenges still remain for the realization of N-doped graphene with low defect densities. First, the mixed flow of carbon and nitrogen precursors in the gas phase triggers a competition between nitrogen incorporation and the NH*_x_* group-based etching effect that induces increased defect densities [23]. The second is the growth temperature-dependent trade-off between the intrinsic defect densities and the nitrogen doping efficiency; while the nitrogen doping efficiency was found to be the largest at low temperatures (600 to 880 °C), the use of these temperatures increases the risk of the formation of intrinsic defects and localized bi-layer or few layer fractions. On the other hand, highly crystallized graphene can be synthesized at elevated temperatures (>950 °C), but the presence of NH*_x_* groups was limited during the growth process [19,25]. The formation of low defect densities in N-doped graphene substantially weakens its induced short-range scattering, but a method for the synthesis of N-doped graphene without transport degradation is still lacking.

To overcome the bottleneck in the direct synthesis of N-doped graphene in the conventional chemical vapor deposition (CVD) process and to obtain high transport properties for N-doped graphene, a solid state doping technique was introduced in this study. The solid state doping technique is broadly used for efficient and uniform doping with low amounts of structural defects in low dimensional materials, such as nanowires and conducting polymers [26,27,28]; however, this technique has rarely been studied experimentally in monolayer graphene decoration [29]. In the current study, Cu foil was chosen as the substrate for monolayer graphene growth owing to its low solubility of carbon. Cu foils were pre-treated by NH_3_ plasma at room temperature, and atomic force microscopy (AFM) was performed to examine the surface morphology variation. This process embeds nitrogen atoms into the Cu substrate, which are subsequently used as the solid-state doping source. Then, N-doped graphene was grown by CVD on the nitrogen-doped copper substrate. X-ray photoelectron spectroscopy (XPS) and Raman spectroscopy were used to analyze the bonding and lattice properties of N-doped graphene. Top-gated graphene field-effect transistors (GFETs) were fabricated using N-doped graphene as the channel, and 1100 cm^2^·V^−1^·s^−1^ carrier mobilities were achieved without carrier mobility degradation, which is comparable to the corresponding pristine graphene.

## 2. Results and Discussion

**Figure 1:**
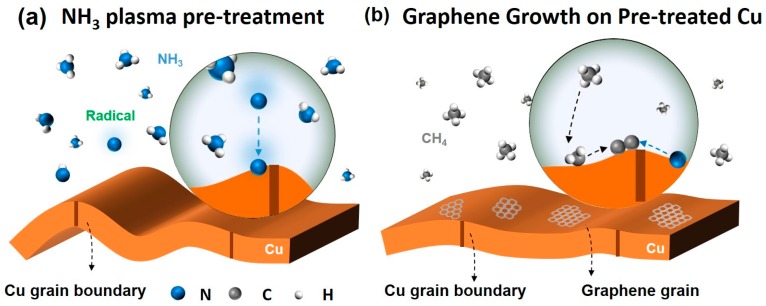
Schematic of (**a**) NH_3_ plasma pretreatment of the Cu surface and (**b**) N-doped graphene synthesis on the pre-treated Cu surface using low-pressure chemical vapor deposition (LPCVD).

Figure 1 shows the illustration of the NH_3_ pretreatment and N-doped graphene synthesis. As illustrated in Figure 1a, during NH_3_ plasma treatment, nitrogen radicals are embedded into the Cu surface, and N–Cu bonds are formed after the surface oxidation and after the organic residues were reduced by hydrogen radicals in the NH_3_ plasma [30,31]. Meanwhile, the Cu surface was flattened during the plasma treatment [32]. The critical point of N–Cu bond formation commonly started after 20 s to 30 s of the NH_3_ plasma treatment [30,31], and a high N/Cu ratio of over 10% could be achieved at the Cu surface [33]. Then, the Cu foils with and without NH_3_ plasma pretreatment were loaded into a low-pressure chemical vapor deposition (LPCVD) chamber for graphene growth separately, as shown in Figure 1b (details are given in the Methods section).

**Figure 2:**
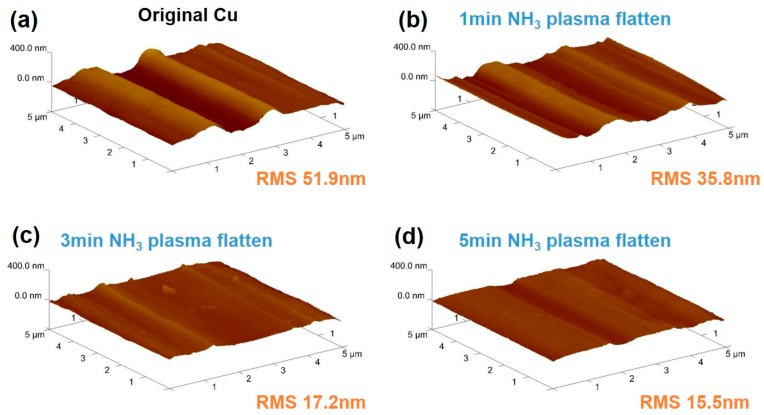
Atomic force microscopy (AFM) images of the Cu surface without and with NH_3_ plasma treatment for different durations: (**a**) original Cu, (**b**) 1 min, (**c**) 3 min, and (**d**) 5 min. RMS is the abbreviation of roughness mean square.

The distinct apparent morphologies of these Cu foil samples with different NH_3_ plasma pretreatment times, acquired in a 5 μm × 5 μm area prior to graphene CVD growth, are shown in Figure 2a–d. The original Cu foil without the NH_3_ plasma pretreatment exhibits a periodically corrugated structure with a maximum height of 202.4 nm and a surface roughness with a root mean square (RMS) value of 51.9 nm. Samples with an NH_3_ plasma pretreatment of 1, 3, and 5 min were labelled as NG1, NG3, and NG5, respectively. The Cu surface morphology becomes less rough as the NH_3_ plasma pretreatment time increases. For the Cu with a 1, 3, and 5 min plasma treatment, the height of the Cu surface corrugation structure and surface roughness decrease to 46.2 nm and 15.5 nm, respectively. The surface-flattening effect of the plasma treatment is comparable to the effects of other Cu pretreatment processes, such as electrochemical polishing or pre-annealing [34]. After the large domain synthesis is complete, graphene and N-doped graphene are transferred onto the SiO_2_ substrate for further measurement and analysis (CVD growth and transfer processes are performed following our previous work, and the details are in the Methods section).

**Figure 3:**
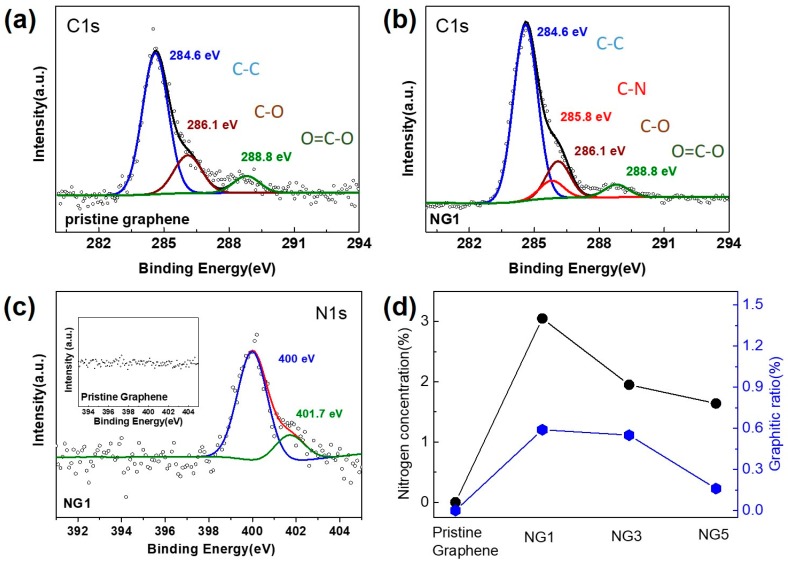
Analysis of X-ray photoelectron spectroscopy (XPS) spectra for pristine graphene and N-doped graphene. (**a**,**b**) C1s spectrum for pristine graphene and NG1; (**c**) N1s core level for NG1 (inset is the N1s spectrum for pristine graphene); (**d**) N content ratio and graphitic configuration ratio for NG1, NG3, and NG5.

To investigate the bonding configurations of N atoms in the graphene lattice, XPS was performed on pristine graphene and N-doped graphene samples after transfer onto the SiO_2_ substrates. The deconvoluted C1s spectra of pristine graphene and NG1 are shown in Figure 3a,b, respectively (Figure S1a,b show the C1s spectra of NG3 and NG5, respectively). The main peak located at 284.6 eV indicates that most C atoms were arranged in the conjugated honeycomb lattice configurations for each sample. In addition to the main peak, peaks at 285.8, 286.1, 287.6, 288.9 eV corresponding to C–N, C–O, C=O, and O–C=O, respectively, were observed; these peaks are attributed to nitrogen doping and organic residues or oxygen absorption. The N1s spectrum is typically used to elucidate the nitrogen dopant configurations in the graphene lattice [35]. As shown in Figure 3c, the N1s spectrum for NG1 can be deconvoluted into two components, pyrrolic N at 400.0 eV and graphitic N at 401.5 eV (detailed N1s spectra for NG3 and NG5 are shown in Figure S1c,d), which is consistent with other CVD approaches (a brief summary of nitrogen doping configurations is given in Table 1). The inset of Figure 3c shows the N1s spectrum of pristine graphene in the N1s spectrum for comparison, demonstrating that the nitrogen doping in the graphene lattice originated from the NH_3_ plasma pretreatment on the Cu surface. Note that the hydrogen annealing process may remove some surface nitrogen atoms. However, based on these results, the amount of surface nitrogen is sufficient for the graphene dopants. As shown in Figure 3d, the nitrogen concentrations of N-doped graphene were 3.05%, 1.95%, and 1.64%, for samples NG1, NG3, and NG5, respectively, which was partially contributed by graphitic N with values of 0.59%, 0.55%, and 0.16%, respectively. The discrepancy between the decreased doping efficiency trend and the increased plasma treatment duration could be ascribed to the substrate-flattening effect by NH_3_ plasma. According to the recent report on atomic-level scanning tunneling microscopy (STM) analysis, nitrogen atoms are preferentially located at the graphene grain boundaries, while there is no indication of nitrogen in the atomically flat graphene regions [36]. Thus, the opportunity for nitrogen doping in rough Cu surface regions is higher than the opportunity in flat Cu surface regions.

**Figure 4:**
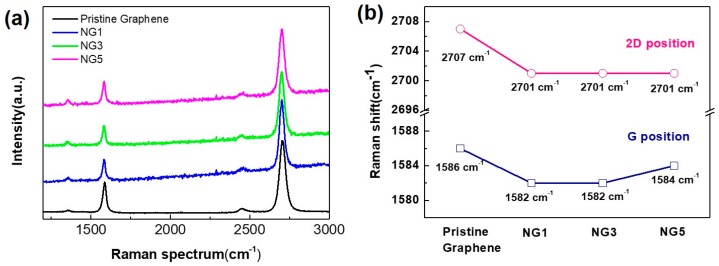
Raman spectra for pristine graphene and N-doped graphene. (**a**) Raman spectrum and (**b**) G and 2D band positions (derived from the Raman spectra).

N-doped graphene samples were also analyzed using their Raman spectra in the 1200–3000 cm^−1^ range as shown in Figure 4. There are four typical graphene vibrational modes in the Raman spectrum: (a) a D peak at 1350 cm^−1^ (defect mode), (b) a G peak at 1586 cm^−1^ (vertical vibration mode), (c) a D’ peak at 1620 cm^−1^ (disorder mode), and (d) a 2D peak at 2707 cm^−1^ (two-phonon vibration mode). The locations of the G peak and the 2D peak of pristine graphene and N-doped graphene are shown in Figure 4a,b. Compared to pristine graphene, N-doped graphene samples (NG1, NG3, and NG5) show a downshift of the G peak from 1586 to 1582, 1582, and 1584 cm^−1^, respectively, and a downshift of the 2D peak from 2707 to 2701, 2701, and 2701 cm^−1^, respectively, indicating n-type doping from pristine graphene to NG1, NG3, and NG5 [37]. The *I*_2D_/*I*_G_ ratios for both pristine graphene and N-doped graphene were greater than 2, as shown in Figure 4a, indicating the high quality and crystallinity of the samples. Derived from the data shown in Figure 4a, the *I*_D_/*I*_G_ ratios of pristine graphene and N-doped graphene range from 0.168 to 0.171, 0.173, and 0.191. Based on the *I*_D_/*I*_G_ ratio, the defect density for each sample can be calculated using the average defect distances based on the Tuinstra-Koenig (TK) relation of [2] (2.4 × 10^−10^)λ^4^(*I*_D_/*I*_G_)^−1^ as 71.4 nm (pristine graphene), 70.1 nm (NG1), 69.3 nm (NG3), and 62,8 nm (NG5). The slightly increasing trend of defect mode intensity is consistent with our XPS survey because more structural defects are induced by pyrrolic N than graphitic N. Moreover, the D’ peak (intra-valley double resonance, which is activated by defects) is weak compared to the G peak, indicating that a relatively low disorder accompanies the nitrogen doping [38]. The incorporation of pyrrolic N is always accompanied by the appearance of structural defects. Pyrrolic N bonds to two sp^2^-hybridized carbon neighbors with adjacent carbon vacancies. In addition, nitrogen incorporation may influence and even interact with the defect formation process during the kinetic growth of graphene because the nitrogen atoms are predominantly distributed at grain boundary regions where defects and vacancies are also easily formed to release the interfacial stress originating from the lattice mismatch between Cu and graphene [36]. The interaction between the released interfacial stress and nitrogen incorporation is reduced by the current pretreatment approach because the pre-existing nitrogen atoms on the Cu surface do not mix with the CH_4_ gas in the processes of gas phase deposition, molecular decomposition, and long-range Cu surface kinetic diffusion.

For comparison, the same plasma treatment process was directly applied to the graphene samples after the transfer onto SiO_2_ substrates. The graphene samples without and with NH_3_ plasma in 1, 3, and 5 min treatments were also examined by Raman spectroscopy, and the Raman spectra are shown in Figure S2. Under 1 and 3 min NH_3_ plasma treatments, strong lattice structural damage was found in which splitting was observed for the D and G peaks, and the 2D peak showed an obvious decrease. For the graphene samples under a 5 min NH_3_ plasma treatment, the typical vibration modes of graphene (D, G, and 2D) almost disappeared. During the NH_3_ plasma treatment directly on graphene, high additional plasma energy after nitrogen displacement remained in the graphene lattice and transferred into in-plane collisions [22]. Thus, the lattice structure was severely damaged as the treatment time increased.

**Figure 5:**
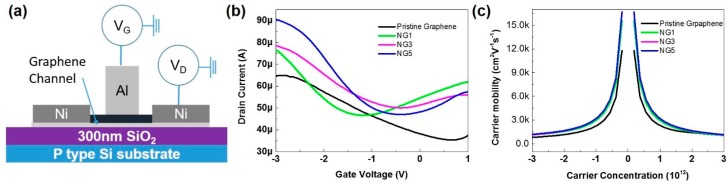
Transport characteristics for pristine graphene and N-doped graphene. (**a**) Schematic structure of top-gated graphene field-effect transistors (GFETs) (**b**) *I*_d_–*V*_g_ curves for pristine, NG1, NG3, and NG5 FETs, with the drain voltage maintained at 0.1 V and a gate voltage sweep from −2.5 to 1 V; and (**c**) Mobility as a function of carrier density for pristine graphene, NG1, NG3, and NG5 FETs.

Finally, to measure the electrical properties, a top-gated GFET was fabricated, as shown in Figure 5a (the details of the fabrication process are given in the Methods section). The source and drain metal contact created by Ni can form an ohmic contact with graphene, as clearly shown in the *I*_d_*V*_d_ curves in Figure S3b. The contact resistivity between Ni and CVD graphene was determined using the transfer length method in the range of 0.7 to 7 kΩ·µm [39,40,41]. Based on this range, the contact resistance could be 1.2% to 12% of the total resistance. Aluminum oxide was chosen as the gate dielectric owing to its high isolation property and low charge transfer effects with graphene (Figure S3a). Figure 5b shows the drain current versus gate voltage curves of each graphene transistor under a gate sweep from −2.5 V to 1.5 V and with the drain voltage fixed at 0.1 V. The Dirac point of pristine graphene was located at 0.7 V, and for the N-doped graphene samples, Dirac points were n-type shifted to −1.2 V (NG1), −0.4 V (NG3), and −0.2 V (NG5). The trend of the Dirac point shift is consistent with the N-doping efficiency, as revealed by the XPS survey. Both pyrrolic and graphitic N were attributed to n-type doping behaviors [20]. In graphitic and pyrrolic N-doping configurations, three valence electrons of the nitrogen atoms are necessary to form bonds with their carbon neighbors, and the remaining two valence electrons provide additional charges to the conduction band, giving rise to n-type doping. Based on the *I*_d_*V*_g_ measurement results and the capacitance of graphene transistors shown in Figure 5b and Figure S3c, the carrier mobility μ of each sample can be derived in terms of the Drude formula ($\mu ={\left(ne\rho \right)}^{-1}$) as a function of the carrier density n (n = *C*_g_(*V*_g_ − *V*_Dirac_)/e), where ρ is the resistance and *C*_g_ is the gate dielectric capacitance [42]. As shown in Figure 5c, the hole mobilities of pristine graphene, NG1, NG3, and NG5 were 809, 1131, 1134, and 1201 cm^2^·v^−1^·s^−1^ (at 3 × 10^12^/cm^2^), respectively. The electron mobilities of pristine graphene, NG1, NG3, and NG5 were 1060, 1061, 1121, and 1151 cm^2^·v^−1^·s^−1^ (at 3 × 10^12^/cm^2^), respectively. The opposite ambipolar mobility behaviors of pristine graphene and N-doped graphene can be clarified using Novikov’s calculation showing that attractive scattering is dominant relative to the repulsive scattering for Dirac fermions in graphene [43]. Therefore, for N-doped graphene, the dominant scattering centers are positive ions, while for the p-type pristine graphene, the asymmetry is different from the N-doped graphene samples. Moreover, a higher carrier mobility and conductivity is shown by N-doped graphene than by pristine graphene. These N-doped graphene samples were synthesized on a pre-treated, flattened Cu surface, while pristine graphene was grown on the original Cu surface. The flattened Cu foil weakens the ripple effect of graphene because graphene grows along the apparent contour of the Cu surface [21,34]. For comparison, the FET of the graphene under 1, 3, and 5 min NH_3_ plasma treatments was also fabricated, and no *I*_d_–*V*_g_ curves were obtained because the graphene was severely damaged during the direct high-energy plasma treatment.

A brief comparison of the N-doped graphene with different CVD synthesis approaches is presented in Table 1, including the parameters of the synthesis conditions, nitrogen concentrations and configurations, *I*_D_/*I*_G_ ratios, Dirac points, and carrier mobilities. Starting with exfoliated graphene, followed by ion implantation and high-temperature NH_3_ annealing, N-doped graphene exhibits a high mobility, reaching 6000 cm^2^·V^−1^·s^−1^. However, this post-annealing doping method for pristine graphene results in a relatively low doping concentration, possibly due to the low probability of graphene interface hybridization between nitrogen atoms and the continuous atomic lattice structure [2,23]. For CVD graphene, which could be manipulated in large-area electronic applications, the N-doping concentration can be adjusted from 2.4% to 16% by precursors or by synthesis conditions. Nitrogen dopant incorporated into the graphene lattice via CVD is mostly found in pyrrolic and pyridinic configurations, which are always accompanied by an increase in defect densities and an increased *I*_D_/*I*_G_ ratio to values ranging from 0.3 to 2 (few-layered N-doped graphene shows a lower *I*_D_/*I*_G_ due to a high vertical phonon vibration nature). Both nitrogen incorporation (ionic impurities, long-range scattering) [44] and its accompanied increase in defect densities (bonding disorder and vacancies, short-range scattering) [45] degrade graphene carrier conductivity and mobility [20]. Recently, George Sarau et al. realized N-doped graphene with a low defect density via the post-annealing approach in which the nitrogen atoms are mostly arranged in pyridinic configurations [23]. Compared with pyrrolic nitrogen, pyridinic nitrogen shows a higher similarity to the original graphene lattice; however, a higher formation energy and synthesis temperature are required for the process [19]. In this study, low defect density N-doped graphene with a pyrrolic dominated nitrogen configuration achieved by combining CVD and the solid source doping technique is proposed. First, low defect density reduces short-range scattering in graphene. Second, the flat Cu surface reduces height fluctuations for 2D graphene [21,34], compensating for the long-range scattering caused by ionic nitrogen incorporation. The current carrier mobility of N-doped graphene is comparable to that found in previous studies: 200–450 cm^2^·v^−1^·s^−1^ (CVD with a mixed gas precursor) [35], 100–400 cm^2^·v^−1^·s^−1^ (plasma enhanced CVD-grown) [17], 365 cm^2^·v^−1^·s^−1^ (pyridine annealing on Cu) [46], and 1050 cm^2^·v^−1^·s^−1^ (substrate doping) [37]. Note that the current study is based on top-gated transistor structures rather than the traditional back-gated graphene transistors using thick SiO_2_ as the gate dielectric, which is more compatible with the integrated circuit (IC) architectures and operates in the low gate voltage mode, but the trade-off is the series resistance in the access region.

## 3. Conclusions

In summary, a CVD synthesis process for low defect density N-doped graphene based on the NH_3_ plasma pretreatment of the Cu foil catalyst was proposed. Approximately 80% of the nitrogen dopant atoms in the graphene lattice are present in the pyrrolic configuration, and the nitrogen concentration was negatively correlated with Cu surface roughness. For synthesis on the pre-treated Cu catalyst, N-doping does not induce a high density of defects or disorder in the graphene lattice, as revealed by the Raman spectra. The electrical transport measurement shows that N-doping effectively modulates the electrical properties of graphene to n-type behavior with high conductivity and carrier mobility. This study provides a low defect density approach for N-doped graphene synthesis and significantly advances the modulation and improvement of the performance of graphene in nanoelectronic applications.

## 4. Methods

### 4.1. NH_3_ Plasma Pretreatment and AFM Scanning

Cu foil (99.8% purity, 25 μm, Alfa Aesar, Ward Hill, MA, USA) was loaded into the plasma-enhanced CVD (PECVD) system, and the chamber was evacuated to 2 mTorr at room temperature. NH_3_ flowed into the chamber at 20 sccm while the pressure was maintained at 0.5 Torr, and radio frequency power of 100 W was applied to generate the NH_3_ plasma. The degree of plasma treatment of each sample was adjusted by controlling the exposure time (from 1 min to 5 min) after the plasma was ignited. The Cu surface morphology was scanned using an Innova AFM (Bruker Company, Billerica, MA, USA) equipped with an Olympus AC160TS probe (tip radius of 7 nm, Kuboyama-cho, Hachioji-shi, Tokyo, Japan). The AFM tip was operated in tapping mode and set to map a 5 × 5 μm^2^ area across the samples.

### 4.2. Graphene Growth and Transfer Process

Copper foils with and without NH_3_ plasma pretreatment were loaded into a low-pressure tube furnace separately [50]. The growth temperature was set to 1000 °C under a H_2_ flow rate of 50 standard cubic centimeters per minute (sccm, cm^3^/min) to remove the native Cu oxide. Then, 20 sccm CH_4_ was introduced to grow graphene on the Cu foils for 20 min. After the graphene synthesis was complete, the furnace was cooled to room temperature at a rate of ~5 °C/s. A layer of polymethyl methacrylate (PMMA) was spin-coated onto the graphene on the Cu surface. The films were then treated with a Cu etchant and rinsed with deionized (DI) water before they were transferred to the 300 nm SiO_2_/Si substrates. The PMMA support layer was removed from each film using acetone, and the remaining graphene films were rinsed with isopropyl alcohol.

### 4.3. Raman Spectral Analysis and X-ray Photoelectron Spectroscopy

Raman spectra were collected using a RAMaker confocal Raman microscope system (PROTRUSTECH, east district, Tainan, Taiwan, laser excitation photon energy of ~2.62 eV; laser spot-size of ~0.5 μm). The Raman peak of Si at 520 cm^−1^ was used as the calibration signal for each sample. For Raman probing, each sample was exposed for 3 s, which was repeated 3 times to obtain an average value and to eliminate unintentional cosmic ray effects. The chemical configurations were determined using an X-ray photoelectron spectrometer (VG Scientific, Microlab 350, East Grinstead, UK) with an Auger electron beam gun (LEG 300-F electron column). The energies were calibrated relative to the C1s peak to eliminate the charge distribution of the sample during analysis.

### 4.4. Top-Gated Graphene Field-Effect Transistors Fabrication and Measurement

After transferring the graphene onto the 300 nm SiO_2_/Si substrate, 50 nm of Ni was deposited as the source and drain (100 μm × 100 μm). The graphene channel (20 µm in width and 50 µm in length) was patterned using lithography methods followed by an O_2_ plasma etching step; finally, 1500 nm of Al was deposited followed by a 1.5-nm Al deposition as a buffer layer. Both of these Al layers were exposed to air for more than 8 h to form a thin AlO*_x_* dielectric layer between the graphene channel and the Al gate [51]. Graphene transistors were measured using a B1500-Agilent semiconductor analyzer (Santa Clara, CA, USA).

## Figures and Tables

**Table 1:** Comparison of N-doping and the electrical performance for N-doped graphene obtained using different growth approaches.

N-Doped Graphene Synthesis Method	Synthesis Temperature	Nitrogen Content ^(^*^)^	Nitrogen Configurations ^(^#^)^	*I*_D_/*I*_G_	Dirac Point Shift	Carrier mobility N-Doped Graphene/Pristine Graphene (cm^2^·V^−1^·s^−1^)	Ref.
CVD monolayer graphene growth on NH_3_ plasma pre-treated Cu foil	1000 °C	3%	Pyrrolic, Graphitic	0.17	0.7 to −1.2 V (top gate)	~1100/~1000 (electron branch) ~1100/800 (hole branch)	This work
CVD monolayer graphene growth on Cu using C_2_H_2_, H_2_ and NH_3_ as precursors	900 °C	16%	Pyridinic	>2	N/A	N/A	[47]
CVD monolayer graphene growth on Cu using CH_4_ and NH_3_ as precursors	1000 °C	6.4%	Pyrrolic	~0.7	N/A	N/A	[48]
CVD monolayer graphene growth on Cu (CH_4_ + NH_3_)	800 °C	8.9%	Graphitic	~0.30	N/A	450/1200	[35]
CVD few-layered (2–8 layers) graphene growth on Ni (CH_4_ + NH_3_ + Ar)	1000 °C	4%	Pyrrolic, Pyridinic	0.06–0.25	N/A	N/A	[49]
CVD few-layered graphene growth on Ni with embedded nitrogen	1000 °C	2.9%	Pyrrolic, Pyridinic, Graphitic	2.1	more than 60 to −50 V (back gate)	N/A	[29]
CVD on Cu using pyridine as the precursor	1000 °C	2.4%	Pyridinic, Pyrrolic	0.3–0.4	10 to −10 V (back gate)	5/2000	[9]
PECVD growth of NG on SiO_2_/Si using C_2_H_2_ and NH_3_ plasma as precursors	475 °C	N/A	Pyridinic	~0.7	20 to −20 V (back gate)	400/NA	[17]
Post-annealing of CVD graphene (on Cu foil) in NH_3_ gas	850 °C	0.25%	Pyrrolic, Pyridinic	~1	N/A	N/A	[7]
Exfoliated graphene with N_2_ ion implantation and post-annealing in NH_3_	1100 °C	1.1%	Pyridinic	~0.6	~2 V to ~−7 V (back gate)	6000/6700 (electron branch) 6000/15000 (hole branch)	[2]

(*) Only the highest nitrogen content samples were chosen in each study; (#) The nitrogen configurations were sequenced by the nitrogen content ratio for each study. The nitrogen pyridinic, pyrrolic, and graphitic configurations accounted for a binding energy of approximately 398.2, 400.3, and 401.5 eV, respectively. CVD = chemical vapor deposition. PECVD = plasma-enhanced CVD.

## Supplementary Materials

The following are available online at http://www.mdpi.com/2079-4991/7/10/302/s1, Figure S1: XPS spectra for N-doped graphene. (a,b) C1s core level for NG3 and NG5; (c,d) N1s core level for NG3 and NG5. Figure S2: Raman spectrum of graphene without and with a 1 min, 3 min, and 5 min NH_3_ plasma treatment. Figure S3: (a) The *I*_g_/*V*_g_ curve of pristine graphene and N-doped graphene; (b) the *I*_d_/*V*_d_ curve of pristine graphene and N-doped graphene; (c) gate capacitance of pristine graphene and N-doped graphene, the inset illustration shows the structure of the capacitance measurement.
