# Supplementary material for: N-Doped Graphene with Low Intrinsic Defect Densities via a Solid Source Doping Technique
Source: Nanomaterials (Basel). 2017 Sep 30;7(10):302. doi: 10.3390/nano7100302 (PMC5666467; doi:10.3390/nano7100302)
Supplement: Supplementary file 1 [file nanomaterials-07-00302-s001.pdf]

Supporting Information

Article

N-doped Graphene with Low Intrinsic Defect Densities via a Solid Source Doping Technique

Bo Liu [1,2,†], Chia-Ming Yang [2,3,4,5,†], Zhiwei Liu [1] and Chao-Sung Lai [2,4,6,7,*]

[1] State Key Laboratory of Electronic Thin Films and Integrate Devices, University of Electronic Science and Technology of China, Chengdu 610054, China; ziv_liu@hotmail.com
[2] Department of Electronic Engineering, Chang Gung University, Taoyuan, 33302, Taiwan; doctorliu8963@gmail.com (B.L.); cmyang@mail.cgu.edu.tw (C.-M.Y.)
[3] Institute of Electro-Optical Engineering, Chang Gung University, Taoyuan, 33302, Taiwan
[4] Biosensor Group, Biomedical Engineering Research Center, Chang Gung University, Taoyuan, 33302, Taiwan
[5] Department of General Surgery, Chang Gung Memorial Hospital, Linkou, 33305, Taiwan
[6] Department of Nephrology, Chang Gung Memorial Hospital, Linkou, 33305, Taiwan
[7] Department of Materials Engineering, Ming Chi University of Technology, New Taipei city, 24301, Taiwan
* Correspondence: cslai@mail.cgu.edu.tw; Tel.: +886(03)2118800#5751
† These authors contributed equally to this work.

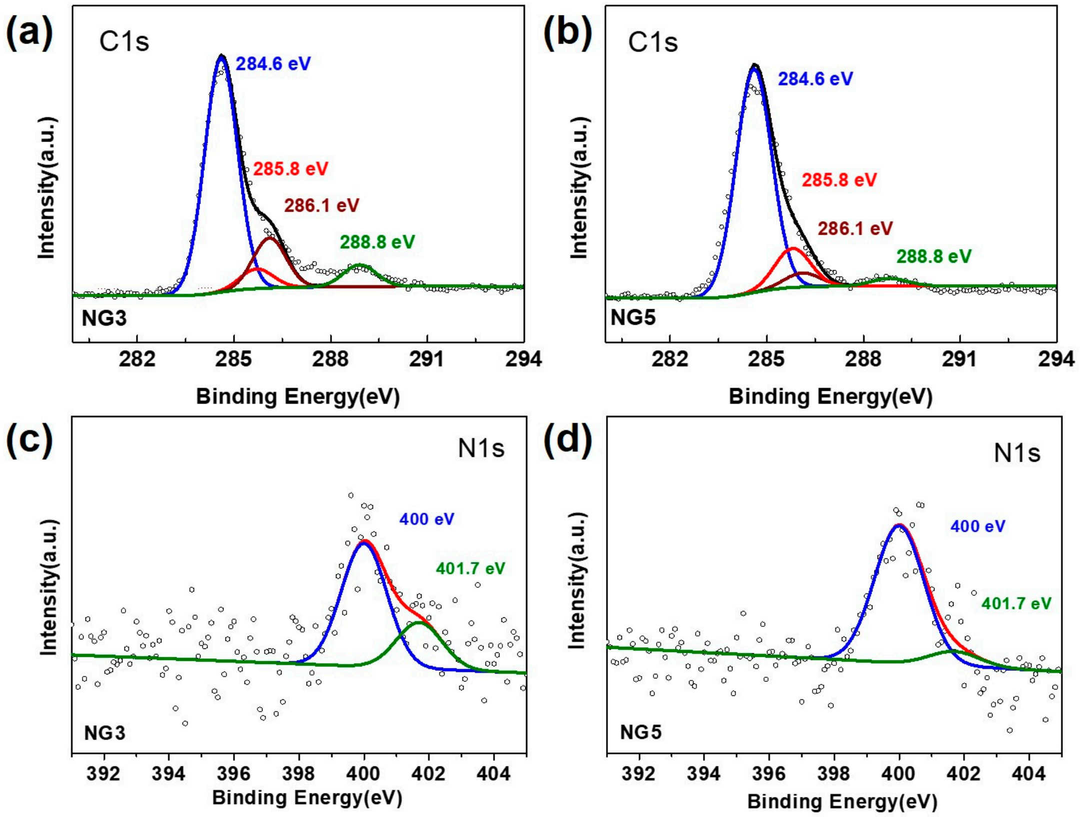

Figure S1. XPS spectra for N-doped graphene. (a-b) C*1s* core level for NG3 and NG5; (c) N*1s* core level for NG3 and NG5.

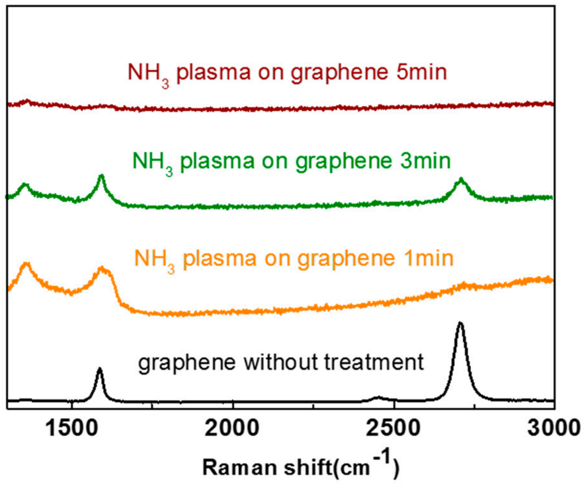

Figure S2. Raman spectrum of graphene without and with a 1 min, 3 min, and 5 min NH_3 plasma treatment.

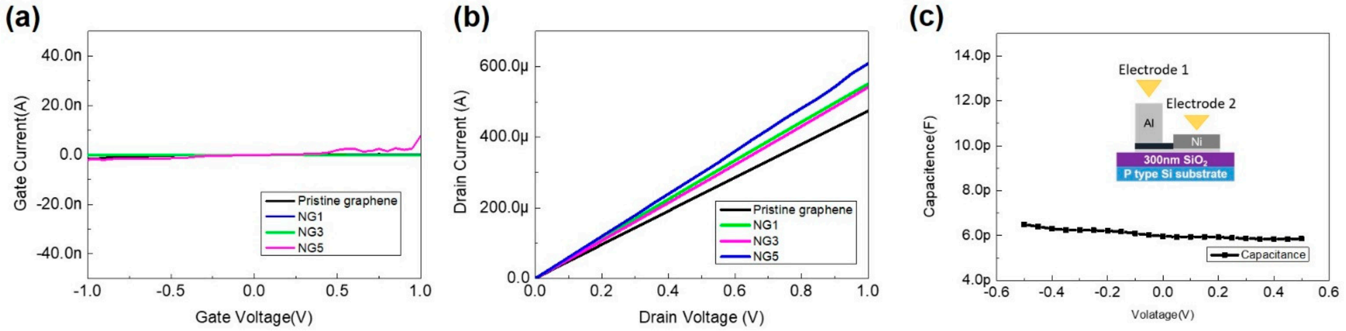

Figure S3. (a) The I_g/V_g output characteristic curve of pristine graphene and N-doped graphene; (b) the I_d/V_d curve of pristine graphene and N-doped graphene; (c) gate capacitance of pristine graphene and N-doped graphene, the inset illustration shows the structure of the capacitance measurement.
